# Pharmacovigilance for single low-dose primaquine: a practical approach

**DOI:** 10.1186/1475-2875-13-S1-P72

**Published:** 2014-09-22

**Authors:** Eugenie Poirot, Andy Stergachis, Feiko ter Kuile, Philippe J Guerin, Ingrid Chen, Roland Gosling

**Affiliations:** 1 Global Health Group, University of California San Francisco, San Francisco, California, USA; 2 Department of Epidemiology and Biostatistics, University of California San Francisco, San Francisco, California, USA; 3 Departments of Epidemiology and Global Health, University of Washington, Seattle, Washington, USA; 4 Department of Clinical Sciences, Liverpool School of Tropical Medicine, Liverpool, UK; 5 Worldwide Antimalarial Resistance Network (WWARN), University of Oxford, Oxford, UK; 6 Centre for Tropical Medicine, Nuffield Department of Clinical Medicine, University of Oxford, Oxford, UK

## 

Establishing and strengthening pharmacovigilance in resource-limited settings provides a valuable opportunity to identify, quantify and address pertinent safety concerns regarding the use of single low-dose (SLD) primaquine. While it is understood that primaquine causes dose-dependent hemolysis in glucose-6-phosphate dehydrogenase (G6PD) deficient individuals with *P. falciparum* and *P. vivax* malaria, the extent of this risk when using low doses of primaquine remains to be defined. Prospective pharmacovigilance methods are needed to confirm or refute these safety concerns that have likely hindered the uptake of the new WHO policy for SLD primaquine.

Pharmacovigilance in resource-limited settings is challenging, especially when attempting to identify unknown or poorly understood adverse drug reactions over weeks of follow-up. In contrast, the objectives of a pharmacovigilance program for SLD primaquine are specific, measurable, and attainable within a short time frame. The nadir of primaquine-induced hemolytic anemia, measurable by hemoglobin concentrations, has been shown to occur on or near day 7. Thus, it is expected that adverse drug reactions related to primaquine can be captured within a week of drug administration by active surveillance methods involving patient follow-up. Furthermore, dark urine is a characteristic sign of acute hemolytic anemia, an easily identifiable and documentable symptom of hemolysis.

Numerous pharmacovigilance efforts are ongoing, supporting individual countries in the collection of standardized SLD primaquine safety data. These efforts seek to establish the expected fall in hemoglobin from baseline levels before treatment to levels seven days post-treatment and include G6PD testing and recording of adverse events using standard data collection instruments. Each participating in-country program or study site aims to collect data on 100-400 subjects depending on malaria endemicity and expected G6PD prevalence. We plan to collate data across these countries in a global database of individual patient data, contributing to the evidence-base for benefit-risk assessments of SLD primaquine.

As other 8-aminoquinoline drugs such as tafenoquine come to market, we will be faced with related safety concerns of dose-dependent hemolysis in G6PD-deficient individuals. Active pharmacovigilance and the establishment of a standardized database for SLD primaquine not only provide opportunities to harmonize efforts, address unanswered questions regarding the safe use of SLD primaquine, and assist programs planning widespread roll-out of SLD primaquine in routine malaria case management they can also serve as a common platform for other current and future pharmacovigilance needs. The methods used in these efforts and the composition of this database will be described.

